# Association between Alcohol Consumption and Health-Related Quality of Life among Hospital and Ambulatory Care Patients with Past Year Depressive Symptoms

**DOI:** 10.3390/ijerph192214664

**Published:** 2022-11-08

**Authors:** Kristian Krause, Diana Guertler, Anne Moehring, Anil Batra, Sandra Eck, Hans-Jürgen Rumpf, Gallus Bischof, Maresa Buchholz, Ulrich John, Christian Meyer

**Affiliations:** 1 Evangelic Hospital Bethania, Gützkower Landstraße 69, 17489 Greifswald, Germany; 2 Department of Prevention Research and Social Medicine, Institute for Community Medicine, University Medicine Greifswald, Walther-Rathenau-Straße 48, 17475 Greifswald, Germany; 3 DZHK (German Center for Cardiovascular Research), Partner Site Greifswald, Fleischmannstraße 42-44, 17489 Greifswald, Germany; 4 Department of Psychiatry and Psychotherapy, University Hospital of Tübingen, Geissweg 3, 72076 Tübingen, Germany; 5 Research Group S:TEP, Department of Psychiatry and Psychotherapy, University of Lübeck, Ratzeburger Allee 160, 23583 Lübeck, Germany; 6 German Center for Neurodegenerative Diseases (DZNE), Site Rostock/Greifswald, Ellernholzstraße 1-2, 17489 Greifswald, Germany

**Keywords:** depression, alcohol consumption, health-related quality of life, VR-12, intervention development

## Abstract

Background: Little is known about how substance use affects health-related quality of life (HRQOL) in depressed individuals. Here, associations between alcohol consumption and HRQOL in hospital and ambulatory care patients with past-year depressive symptoms are analyzed. Method: The sample consisted of 590 participants (26.8% non-drinkers) recruited via consecutive screenings. Individuals with alcohol use disorders were excluded. HRQOL was assessed with the Veterans Rand 12-item health survey (VR-12). Multivariable fractional polynomials (MFP) regression analyses were conducted (1) to test for non-linear associations between average daily consumption and HRQOL and (2) to analyze associations between alcohol consumption and the physical and mental health component summaries of the VR-12 and their subdomains. Results: Alcohol consumption was positively associated with the physical health component summary of the VR-12 (*p* = 0.001) and its subdomains general health (*p* = 0.006), physical functioning (*p* < 0.001), and bodily pain (*p* = 0.017), but not with the mental health component summary (*p* = 0.941) or any of its subdomains. Average daily alcohol consumption was not associated with HRQOL. Conclusion: Alcohol consumption was associated with better physical HRQOL. Findings do not justify ascribing alcohol positive effects on HRQOL. Data indicate that non-drinkers may suffer from serious health disorders. The results of this study can inform the development of future alcohol- and depression-related interventions.

## 1. Introduction

Health-related quality of life (HRQOL) is a psychological construct describing physical, mental, social, psychological, and functional aspects of well-being and function from the perspective of patients [1]. Depressive disorders are associated with impairments in HRQOL (e.g., [2,3,4]), even in individuals without clinically relevant depressive symptoms [5] or those who remitted from depression [6]. Previous studies on how alcohol consumption may affect the quality of life in depressed individuals have produced inconclusive results. Danovitch et al. [7] did not find differences in quality of life and functioning between individuals with major depressive disorders without an alcohol use disorder (AUD) and depressed patients with an AUD. Contradictory to this, Levola et al. [8] found alcohol problems to be associated with decreases in quality of life in depressed individuals, and a systematic review by Sullivan et al. [9] concluded that alcohol problems in depressed individuals are associated with reduced social functioning. While the overall number of studies examining the association between alcohol problems and HRQOL in depressed individuals is small, the effects of alcohol on HRQOL were extensively studied in other samples. These show that in individuals with AUDs, particularly alcohol dependence, alcohol leads to significant impairments in quality of life (e.g., [10,11,12]). However, for less severe consumption patterns, extensive data hints at positive effects of alcohol consumption on HRQOL (e.g., [13,14,15,16,17]). Some studies further provided evidence for non-linear associations between alcohol consumption and HRQOL [18,19,20,21,22]. According to these findings, HRQOL increases up to a certain consumption level or severity level of alcohol problems before it starts to decrease. Such findings are consistent with a repeatedly demonstrated J-curved association between alcohol consumption and health and mortality outcomes which led to the conclusion that drinking alcohol in small amounts may have beneficial health effects [23,24,25]. However, the plausibility of corresponding results and the conclusions drawn from them have long been questioned [26]. Meta-analytical data show that findings from which a potential health benefit of drinking alcohol in small amounts has been concluded are likely to be the consequence of a methodological artifact known as the abstainer bias. It is caused by the misclassification of former and occasional drinkers, who often suffer from ill health, as abstainers [27,28]. A recent longitudinal study by John et al. [29] provided further evidence for this notion. In a random general population sample, they showed that among alcohol abstainers, many former drinkers are to be found. In addition, the majority of alcohol abstainers carried risk factors that increased their likelihood of premature death.

The aim of this cross-sectional study was to analyze how alcohol consumption is associated with HRQOL in a consecutive sample of hospital and ambulatory care patients with past-year depressive symptoms. This group may be considered morbid in its own right. However, based on the aforementioned literature, we suspect that alcohol-abstinent hospital and ambulatory care patients with past-year depressive symptoms have particularly high morbidity and thus report worse HRQOL compared to those consuming alcohol.

## 2. Materials and Methods

### 2.1. Participants

The sample for this analysis comes from two randomized controlled trials testing the efficacy of computer-based interventions targeting either depressive symptoms and accompanying heavy alcohol consumption (study 1) or depressive symptoms alone (study 2). From January 2017 to March 2018, general hospital and general medical practice patients aged 18–64 were systematically screened for depressive symptoms and heavy consumption patterns on three sites across Germany. A detailed description of the screening procedure is provided elsewhere [30]. To be eligible for study 1, patients must have suffered at least one episode with at least subclinical depressive symptoms during the 12 months prior to the screening and must have practiced heavy drinking. The depression-related inclusion criterion applied if, within a period of at least 2 weeks, at least 2 DSM-IV [31] symptoms for major depression, including depressed mood or anhedonia, were present on more than half of the days [32]. Heavy drinking was defined as drinking more than 12/24 g (women/men) of pure alcohol per day on average and/or practicing binge drinking, defined as drinking 4/5 (women/men) drinks or more per occasion at least once a month. For study 2, only the depression-related inclusion criterion had to be present. Participants were not eligible if a current severe depressive episode and/or a moderate or severe AUD had to be suspected.

In total, 12,828 patients provided complete screening data. Of the 331 individuals who were offered to participate in study 1, 168 (50.8%) consented to do so. Participation in study 2 was offered to 1257 individuals, of whom 618 (49.2%) consented. Of those who declared to participate, 134 (79.8%) study 1 and 456 (73.8%) study 2 participants provided sufficient data to be included in the present analyses. Thus, the final sample consisted of 590 individuals. Among them, 158 (26.8%) reported being alcohol abstinent.

### 2.2. Measures

#### 2.2.1. Depressive Symptoms

Depressive symptoms were assessed with the 8-item version of the Patient Health Questionnaire (PHQ-8; [32]). Symptoms can be categorized into 5 severity levels based on the PHQ-8 score: subthreshold (0–4), mild (5–9), moderate (10–14), moderately severe (15–19), and severe (≥20). A PHQ-8 sum score ≥10 is considered clinically relevant [32]. The ascertained sum score referred to the 2 weeks within the past 12 months in which the participant felt worst. If patients reported that depressive symptoms were present during the past two weeks and their PHQ-8 sum score during their worst episode during the past 12 months was ≥20, a current severe depressive episode was suspected, and patients were excluded from either study.

#### 2.2.2. Alcohol Consumption

Drinking behavior was assessed with the Alcohol Use Disorder Identification Test (AUDIT; [33]). This consists of 10 items asking for consumption patterns and experienced negative consequences of alcohol consumption. Each item can be scored from 0 to 4. At an AUDIT score of ≥20, an AUD was assumed [34], and patients were deemed ineligible for study participation. Average daily intake of pure alcohol in grams per day was determined by complementing the categorical AUDIT consumption items by a continuous assessment of alcohol consumption [35] and calculating a quantity-frequency index assuming 10.5 g of pure alcohol for one standard drink. Binge drinking was determined by using a gender-specific version of the AUDIT item 3 considering thresholds defined in the respective inclusion criterion (≥4/5 drinks per occasion for women and men, respectively; [36]). Categorization of drinking status was performed based on the answer to the first AUDIT item, “How often do you have a drink containing alcohol?” This was asked without defining a specific timeframe. Those answering “never” were categorized as non-drinkers, all others as drinkers.

#### 2.2.3. Health-Related Quality of Life

HRQOL was assessed using the German version of the Veterans RAND 12-item health survey (VR-12; [37]). The VR-12 measures eight health domains, which are summarized into a t-standardized physical (PCS) and mental component summary score (MCS). The domains are physical functioning (includes, for example, asking for experienced impairments in daily activities such as vacuuming), social functioning (asks how often social contacts were impaired due to physical or mental health), role limitation due to physical problems (includes, for example, asking how often an individual did manage less than intended due to physical problems), role limitation due to emotional problems (includes, for example, asking how often an individual did manage less than intended due to emotional problems), mental health (includes, for example, asking about the frequency of feelings of discouragement or sadness), energy/vitality (asks how often an individual felt full of energy), bodily pain (asks to what extent pain has affected everyday life), and general perception of health (asks for a subjective rating of one’s health). The VR-12 was administered as part of a computer-assisted telephone interview held after patients consented to participate in one of the studies. For items that asked for the individual’s feelings or impairments within a specific timeframe, the timeframe was chosen to be the past week.

Raw data for each item of the VR-12 were transformed to a 0 (worst possible quality of life) to 100 (best possible quality of life) scale before building the PCS and MCS scores with a predefined algorithm. PCS and MCS scores can range from 0 to 100. Higher numbers correspond to better HRQOL. Subscale scores (i.e., scores for each health domain) for each participant corresponded to the transformed raw item score. For health domains that are measured with 2 items, subscale scores for each participant corresponded to the mean of the transformed raw item scores.

### 2.3. Statistical Analyses

Calculations were carried out using the Stata 14 (Version 14.2) statistical software package [38]. Group differences in terms of sociodemographics, depressive symptoms, and alcohol consumption were calculated using independent t-tests (continuous data) and chi-square tests (categorical data). To analyze the association between HRQOL and alcohol consumption in hospital and ambulatory care patients with past-year depression, we applied the multivariable fractional polynomial approach (MFP; [39]) using Stata’s *mfp* command. This approach allows checking for complex non-linear associations between the average daily alcohol consumption and HRQOL. Regression model building was performed under the assumption that individuals who drink alcohol qualitatively differ from those who do not. To account for that assumption, the *catzero* option was applied to the continuous predictor *average daily consumption*. By doing so, MFP (1) automatically created a binary variable for alcohol consumption (drinker vs. non-drinker) and included it in the model, and (2) treated both the binary and continuous consumption variables as predictors in the model [40]. MFP allowed to assess (a) whether the consumption of alcohol (yes vs. no) affects HRQOL in general and (b) whether the amount that is consumed is relevant beyond drinking status. MFP analyses were controlled for sociodemographics and recruitment setting. Sociodemographic covariates included age, sex (female vs. male), employment status (unemployment vs. fully or part-time employment), relationship status (not in a committed relationship vs. in a committed relationship), and years of education (>10 years vs. ≤10 years or unknown). For variable selection, the default selection level of 1 was used, forcing all variables into the models. For testing between different fractional polynomial models, the default significance level of α = 0.05 was used. Analyses were explorative. We did not adjust for multiple testing.

## 3. Results

### 3.1. Sociodemographics

The mean age of the sample was 39.3 (SD = 14.0) years (Table 1). Non-drinkers were significantly older than drinkers. Drinking status was further related to education level, employment status, and recruitment setting. Compared to drinkers, fewer non-drinkers had an education of more than 10 years, were employed full- or part-time, and were recruited in medical practices.

### 3.2. Depressive Symptoms

The majority of the sample (71.9%) reported no active depression (i.e., symptoms within the past two weeks) at the time of screening. Drinkers and non-drinkers did not differ in that regard (non-drinkers: 66.5%, drinkers: 73.8%, *p* = 0.077). The mean PHQ-8 score of the worst depressive episode within the past year was 13.9 (SD = 3.8) within the whole sample. No group differences were observed for mean PHQ-8 score (non-drinkers: M = 14.4, SD = 3.5; drinkers: M = 13.7, SD = 3.9; *p* = 0.052), observed depression severity levels (*p* = 0.106), and in terms of the percentage of individuals who experienced clinically relevant depressive symptoms (overall: 86.8%, non-drinkers: 89.9%, drinkers: 85.7%, *p* = 0.180).

### 3.3. Alcohol Consumption

The mean daily consumption level of drinkers was 4.3 (SD = 6.3) grams. The drinking subsample consisted of 134 (31.0%) heavy drinkers recruited for study 1 and 298 (69.0%) occasional drinkers recruited for study 2. All heavy drinkers reported bingeing at least once a month. Drinking above recommended daily levels was reported by 21 (15.7%) heavy drinkers. Of those, 71.4% were female. Heavy drinkers reported significantly higher mean daily consumption levels than occasional drinkers (heavy drinkers: M = 9.2, SD = 8.6; occasional drinkers; M = 2.1, SD = 2.9; *p* < 0.001).

### 3.4. Health-Related Quality of Life

#### 3.4.1. Mean Health-Related Quality of Life Scores among Alcohol Drinkers and Non-Drinkers

Compared to non-drinkers, drinkers reported a better overall physical HRQOL (Table 2). Significant differences were found on all physical HRQOL subscales, with drinkers reporting better general health, better physical functioning, fewer role limitations due to physical problems, and less bodily pain than non-drinkers. Differences between groups were largest for physical functioning, followed by bodily pain and general health perceptions.

Drinkers and non-drinkers did not differ in terms of overall mental HRQOL. However, significant differences were observed on the mental HRQOL subscales *mental health* and *social functioning,* with drinkers reporting better mental health and better social functioning compared to non-drinkers.

#### 3.4.2. Multivariable Fractional Polynomial Analyses

In the MFP regression models controlling for covariates (Table 3), non-drinking was associated with worse overall physical HRQOL, worse general health perceptions, worse physical functioning, and more bodily pain. Daily consumption levels did not show any significant associations with physical HRQOL beyond drinking status. Higher age was consistently associated with worse physical HRQOL (overall physical HRQOL: Coef. = −0.265, SE = 0.034, *p* < 0.001; general health perceptions: Coef. = −0.590, SE = 0.072, *p* < 0.001; physical functioning: Coef. = −0.749, SE = 0.092, *p* < 0.001; role limitations–physical: Coef. = −0.322, SE = 0.088, *p* < 0.001; bodily pain: Coef. = −0.522, SE = 0.104, *p* < 0.001), being employed with better physical HRQOL (overall physical HRQOL: Coef. = 2.343, SE = 0.894, *p* = 0.009; general health perceptions: Coef. = 5.891, SE = 1.917, *p* = 0.002; physical functioning: Coef. = 12.052, SE = 2.438, *p* < 0.001; role limitations–physical: Coef. = 5.445, SE = 2.340, *p* = 0.020; bodily pain: Coef. = 5.579, SE = 2.766, *p* = 0.044). Male sex was associated with fewer role limitations due to physical problems (Coef. = 6.228, SE = 2.417, *p* = 0.010). School education of 10 years or less was associated with worse overall physical HRQOL (Coef. = −2.799, SE = 0.927, *p* = 0.003), worse general health perceptions (Coef. = −8.930, SE = 1.987, *p* < 0.001), and more bodily pain (Coef. = −11.464, SE = 2.868, *p* < 0.001). Furthermore, hospital recruitment was associated with worse overall physical HRQOL (Coef. = −6.116, SE = 0.950, *p* < 0.001), worse physical functioning (Coef. = −12.588, SE = 2.591, *p* < 0.001), more role limitations due to physical problems (Coef. = −13.429, SE = 2.487, *p* < 0.001), and more bodily pain (Coef. = −10.299, SE = 2.939, *p* < 0.001).

As for the mental HRQOL, neither drinking status nor daily consumption levels showed associations with the overall mental HRQOL or any of the mental health domains (Table 3). Higher age was associated with worse energy/vitality (Coef. = −0.207, SE = 0.777, *p* = 0.008) and worse social functioning (Coef. = −0.486, SE = 0.102, *p* < 0.001). Being employed was associated with better overall mental HRQOL (Coef. = 2.627, SE = 0.998, *p* = 0.009), fewer role limitations due to emotional problems (Coef. = 8.779, SE = 2.257, *p* < 0.001), and better mental health (Coef. = 5.795, SE = 1.816, *p* = 0.001). Being in a relationship was associated with better overall mental HRQOL (Coef. = 2.109, SE = 1.028, *p* = 0.041). School education of 10 years or less was associated with worse mental health (Coef. = −6.454, SE = 1.881, *p* = 0.001). Furthermore, hospital recruitment was associated with better overall mental HRQOL (Coef. = 4.106, SE = 1.061, *p* < 0.001) and fewer role limitations due to emotional problems (Coef. = 6.800, SE = 2.396, *p* = 0.005).

We did not find any non-linear associations between overall physical or mental HRQOL or any of the physical or mental health domains and average daily alcohol consumption levels (data not presented here).

## 4. Discussion

In this study, we analyzed how alcohol consumption was associated with HRQOL in hospital and ambulatory care patients with past-year depressive symptoms. The main findings are: (1) Non-drinking hospital and ambulatory care patients with past-year depressive symptoms report worse HRQOL compared to drinking hospital and ambulatory care patients with past-year depressive symptoms. (2) Associations between alcohol consumption and HRQOL are restricted to the physical HRQOL and its subdomains. (3) In this sample consisting of individuals with rather low average daily consumption levels, drinking status is associated with physical HRQOL, but the average daily amount of consumption is not. (4) No non-linear associations exist between alcohol consumption and HRQOL in this sample of hospital and ambulatory care patients with past-year depressive symptoms.

Our results are in line with previous cross-sectional analyses reporting better physical but not mental HRQOL in drinking compared to non-drinking individuals [16,17,41] and with cross-sectional and longitudinal studies reporting better HRQOL in connection with alcohol consumption (e.g., [13,14,15,21,42,43]). Associations need to be interpreted with caution. Data do not justify ascribing alcohol positive effects on HRQOL in hospital and ambulatory care patients with past-year depressive symptoms. The fact that associations between consumption and better quality of life were restricted to the physical HRQOL indicates that severe physical health issues existed in the group of non-drinking individuals in our sample. When looking at the mean scores of the physical HRQOL subdomains, non-drinking individuals reported specifically low general health perceptions. The numerically largest group differences were observed according to physical functioning, thus showing much more pronounced physical impairments in non-drinking compared to drinking individuals. The observed mean differences could be related to the significantly higher mean age of the non-drinking subgroup in our sample. Age is known to be a main risk factor for diseases [44], and the deteriorating health that accompanies older age has long been linked to a reduction or cessation of alcohol consumption [26]. However, age differences alone do not explain the observed associations between drinking status and HRQOL, as we controlled for age in our MFP analyses. The fact that mental HRQOL was not associated with alcohol consumption supports the importance of physical disability in explaining the observed associations. The mean differences in the mental HRQOL subdomains *mental health* and *social functioning* could also be explained with physical illness as (a) physical illness is known to be associated with mental health problems [45,46] and (b) the social functioning item is taking physical health into account when asking for impairments in social life. In addition to the arguments mentioned so far, the settings in which participants were recruited also speak in favor of the presumed health impairments in the non-drinking individuals in our sample. Participants recruited in general hospitals and general medical practices can be regarded as in need of treatment for physical illness. Some of these conditions are likely to be accompanied by a physician’s recommendation of alcohol abstinence or even prohibition of use. These cases may be found predominantly in the group of non-drinking individuals. Overall, the data provided here support the assumption that groups of non-drinkers represent particularly morbid groups [29] and that existing health impairments might be the initial reason for choosing abstinence [26]. This sick-quitter phenomenon could also explain why drinking status is associated with better HRQOL in this sample of hospital and ambulatory care patients with past-year depressive symptoms, while average daily consumption is not. It has previously been assumed that regular low-level alcohol consumption might be an indicator of good health but not its cause [28].

In accordance with our assumption, previous cross-sectional and longitudinal research on the association between alcohol consumption and HRQOL repeatedly found results indicative of the sick quitter phenomenon [14,47,48,49]. On the other hand, there are also studies that provide evidence for positive effects of alcohol consumption on HRQOL while seemingly taking the sick quitter phenomenon into account [16,20,41,50]. However, a closer look at the operationalization of drinking status in these studies shows that they are prone to misclassification errors [27]. Lee and Kim [50] categorized individuals as never drinkers who answered “no” to the question “Have you ever had more than one drink of alcohol in your life?”, which allows a regular consumption of not more than one drink to be categorized as a “never drinker”. Ortolá et al. [16] distinguished between non-drinkers and ex-drinkers, but the non-drinking group did include occasional drinkers. The same is true for the study of Valencia-Martin et al. [41]. Schrieks et al. [20] distinguished between former drinkers and abstainers, but abstainers had to abstain from alcohol only in the last eight years.

Misclassifying sick quitters as abstainers and the non-consideration of other health-risk behaviors aside from consuming alcohol are identified as major reasons for the frequently found J-shaped association between alcohol consumption and health and mortality outcomes [27,29], in which low-level alcohol consumption has been interpreted to have beneficial effects on health compared to abstinence and heavy consumption. Some studies provided evidence that comparable non-linear associations exist between alcohol consumption and HRQOL [18,19,20,21]. We did not find such associations within our sample of hospital and ambulatory care patients with past-year depressive symptoms. However, this is not surprising given the low average daily consumption levels of drinkers in this sample. Deteriorating effects of alcohol on quality of life have been shown in consumption patterns representing AUDs (e.g., [10,11,12]). Individuals with corresponding consumption patterns, however, were excluded from our study.

The results of this study and previous work showing the importance of misclassification errors in studies analyzing associations between alcohol consumption and health and mortality outcomes [27,29] provide important implications for the development of future interventions targeting alcohol consumption in large populations (e.g., e-health interventions). Instead of merely focusing on individuals currently consuming alcohol, interventions should also address alcohol-abstinent individuals with previous consumption and provide intervention components aimed at improving quality of life (e.g., [51,52]). Abstinence status could further be considered for tailoring interventions targeting depressive symptoms in large populations. For example, although previous studies show positive effects of exercise on depressive symptoms (e.g., [53,54]), it may not be advised to former drinkers due to their potential critical health.

Previous research on the impact of alcohol consumption on depression mainly focused on individuals with AUDs in psychiatric settings [9]. Being conducted outside the psychiatric setting and analyzing a sample consisting of individuals with lower-level alcohol consumption patterns is a major strength of this study. The associations found in our analyses should not lead to an underestimation of the adverse effects of alcohol consumption on health, even in lower-level alcohol consumption patterns. Apart from AUDs, consuming alcohol is an important risk factor for numerous other health issues, including cancer, diabetes, cardiovascular disease, and liver and pancreas disease [55]. A recent analysis by Schutte et al. [56] has shown that consuming even low amounts of alcohol is associated with an increased risk of all-cause mortality and various negative health outcomes, including cancer, cardiovascular events, and cerebrovascular disease. 

This study has several limitations. First, data are cross-sectional. They do not allow causal inference. Second, all data are self-reported. They could be biased towards social desirability. Furthermore, recall bias has to be considered. Third, we controlled for basic sociodemographic factors but failed to do so for other factors possibly affecting the association between alcohol consumption and HRQOL in individuals with past-year depressive symptoms, including psychiatric and somatic health disorders. Fourth, as individuals with an active severe depression and/or an AUD were excluded, the generalizability of our data is restricted. Fifth, we cannot provide proof for our assumption that the results reflect the sick-quitter phenomenon as we did not have information on why study participants chose to stay alcohol abstinent. Furthermore, our assessment does not allow us to distinguish between former and never drinkers, nor do we know for how long individuals were alcohol abstinent by the time of our assessment. Data collection on individuals drinking status should include asking those who do not drink about the length of their abstinence and about potential reasons for abstaining from alcohol. In doing so, different types of abstainers might be analyzed [29]. This might provide the opportunity to receive more unbiased results on the effects of alcohol on HRQOL [47].

## 5. Conclusions

This study shows better physical HRQOL in drinking compared to non-drinking hospital and ambulatory care patients with past-year depressive symptoms. Our findings do not permit recommending alcohol consumption to promote HRQOL in individuals with past-year depressive symptoms. Data may suggest that non-drinkers in our sample suffer from health problems, which, for the majority of them, might explain staying abstinent from alcohol [26]. Future interventions targeting alcohol consumption and/or depressive symptoms in large populations should consider abstinence status as an important variable when deciding whom to address and what information to provide.

## Figures and Tables

**Table 1:** Sociodemographics.

Variable	All (*n* = 590)	Non-Drinker (*n* = 158)	Drinker (*n* = 432)	*p*
Age, M (SD)	39.3 (14.0)	43.6 (13.7)	37.8 (13.8)	**<0.001**
Sex, %				
Female	363 (61.5)	91 (57.6)	272 (63.0)	0.235
Male	227 (38.5)	67 (42.4)	160 (37.0)	
Education, %				
>10 Y or still in school	285 (48.3)	51 (32.3)	234 (54.2)	**<0.001**
≤10 Y, no degree/uncertain	305 (51.7)	107 (67.7)	198 (45.8)	
Employment Status, %				
Unemployed/student/other	251 (42.5)	83 (52.5)	168 (38.9)	**0.003**
Employed (full- or part-time)	339 (57.5)	75 (47.5)	264 (61.1)	
Marital Status, %				
No current relationship	210 (35.6)	52 (32.9)	158 (36.6)	0.411
In a relationship ^a^	380 (64.4)	106 (67.1)	274 (63.4)	
Setting, %				
Medical Practice	371 (62.9)	87 (55.1)	284 (65.7)	**0.017**
General Hospital	219 (37.1)	71 (44.9)	148 (34.3)	

Note. Bold numbers indicate significant results. ^a^ includes divorced/separated and widowed individuals in new relationships.

**Table 2:** Mean health-related quality of life scores.

	All (*n* = 590)	Non-Drinker (*n* = 158)	Drinker (*n* = 432)	*p*
**Physical component, M (SD)**				
Physical component summary score	37.2 (12.4)	32.0 (12.4)	39.2 (11.9)	**<0.001**
General health perceptions	43.7 (25.6)	34.1 (26.2)	47.6 (24.4)	**<0.001**
Physical functioning	69.9 (33.6)	54.9 (35.3)	75.4 (31.2)	**<0.001**
Role limitations–physical	57.7 (29.5)	50.9 (27.6)	60.2 (29.8)	**<0.001**
Bodily pain	52.8 (35.5)	40.8 (36.7)	57.2 (34.0)	**<0.001**
**Mental component, M (SD)**				
Mental component summary score	37.9 (11.9)	37.5 (12.1)	38.1 (11.9)	0.570
Role limitations–emotional ^a^	61.2 (26.9)	60.6 (27.2)	61.5 (26.9)	0.721
Energy/vitality	35.8 (24.4)	33.3 (25.1)	36.7 (24.1)	0.136
Mental health ^b^	52.7 (21.8)	49.1 (23.4)	54.0 (21.0)	**0.015**
Social functioning	58.6 (33.2)	50.5 (35.9)	61.5 (31.7)	**<0.001**

Note: Bold numbers indicate significant group differences. ^a^ one non-drinker had to be excluded due to a missing on one of the items building the subscale *role limitations due to emotional problems*; ^b^ one non-drinker had to be excluded due to a missing on one of the items building the subscale *mental health.* M, mean; SD, standard deviation.

**Table 3:** Results of the multivariable fractional polynomial regression analyses for the predictors *Non-Drinker* (drinking status) and *Gram/Day* (average daily consumption) for the physical and mental component of the VR-12 and their respective subscales.

	Non-Drinker			Gram/Day		
**Physical component**	**Coef.**	**SE**	***p***	**Coef.**	**SE**	***p***
Physical component summary score	−3.600	1.081	**0.001**	0.101	0.083	0.223
General health perceptions	−6.435	2.317	**0.006**	0.166	0.178	0.352
Physical functioning	−10.552	2.948	**<0.001**	0.377	0.226	0.097
Role limitations–physical	−4.015	2.829	0.156	0.120	0.217	0.581
Bodily pain	−8.009	3.343	**0.017**	0.253	0.257	0.325
**Mental component**	**Coef.**	**SE**	***p***	**Coef.**	**SE**	***p***
Mental component summary score	−0.090	1.206	0.941	0.054	0.093	0.558
Role limitations–emotional ^a^	0.948	2.733	0.729	0.095	0.209	0.651
Energy/vitality	−1.738	2.496	0.487	0.190	0.192	0.322
Mental health ^b^	−2.777	2.195	0.206	0.109	0.168	0.518
Social functioning	−5.534	3.291	0.093	0.258	0.253	0.308

Note. Bold numbers indicate significant results. ^a^ one non-drinker had to be excluded due to a missing on one of the items building the subscale *role limitations due to emotional problems*; ^b^ one non-drinker had to be excluded due to a missing on one of the items building the subscale *mental health.* Coef., coefficient; SE, standard error.

## Data Availability

The data presented in this study are available on request from the corresponding author.
